# Sex-Related Outcome After Sutureless Aortic Valve Replacement With Perceval Plus: Results From a Global Registry and Meta-Regression

**DOI:** 10.1093/icvts/ivag170

**Published:** 2026-06-03

**Authors:** Giuseppe Santarpino, Roberto Lorusso, Michele Di Mauro, Veronica D’Anna, Chiara Maria Prencipe, Pierre Voisine, Gianluigi Bisleri, Eugene Parrino, Michael Bates, G Chad Hughes, Eric Roselli, Cristian Baeza, David Heimansohn, Thierry Bové, Bart Meuris, Herbert Gutermann, Pierre Corbi, Francesco Pollari, George Awad, Utz Kappert, Friedrich Mellert, Sharaf-Eldin Shehada, Evaldas Girdauskas, Giovanni Troise, Marco Solinas, Giuseppe Minniti, Enzo Mazzaro, Mauro Rinaldi, Davide Pacini, Vincenzo Argano, Michele Torella, Ka Yan Lam, Slobodan Micovic, Matthias Siepe, Max Baghai, Seung Hyun Lee, Jae Woong Choi, Hyung Gon Je, Angelo Nobre

**Affiliations:** Department of Experimental and Clinical Medicine, Magna Graecia University, Catanzaro 88100, Italy; Department of Cardiac Surgery, Città di Lecce Hospital, Lecce 73100, Italy; Department of Cardiac Surgery, Paracelsus Medical University, Nuremberg 90419, Germany; Department of Cardiothoracic Surgery, Heart and Vascular Centre, Maastricht University Medical Centre, Maastricht 6229 HX, Netherlands; Department of Medical and Surgical Sciences, University of Foggia, Foggia 71122, Italy; Department of Cardiac Surgery, Città di Lecce Hospital, Lecce 73100, Italy; Department of Experimental and Clinical Medicine, Magna Graecia University, Catanzaro 88100, Italy; Department of Cardiac Surgery, Université Laval, Quebec G1V 0A6, Canada; Department of Cardiac Surgery, St. Michael’s Hospital, Toronto M5B 1W8, Canada; Department of Cardiac Surgery, Ochsner Clinic Foundation, New Orleans, LA 70121, United States; Department of Cardiac Surgery, East Carolina University, Greenville, NC 27858, United States; Department of Cardiac Surgery, Duke University, Durham, NC 27708, United States; Department of Cardiac Surgery, Cleveland Clinic, Cleveland, OH 44195, United States; Department of Cardiac Surgery, University Hospitals Cleveland Medical Center, Cleveland, OH 44195, United States; Department of Cardiac Surgery, St. Vincent Heart Center of Indiana, Indianapolis, IN 46290, United States; Department of Cardiac Surgery, UZ Gent, Gent 9000, Belgium; Department of Cardiac Surgery, UZ Leuven, Leuven 3000, Belgium; Department of Cardiac Surgery, Ziekenhuis Oost Limburg, Genk 3600, Belgium; Department of Cardiac Surgery, CHU Poitiers, Poitiers 86021, France; Department of Cardiac Surgery, Paracelsus Medical University, Nuremberg 90419, Germany; Department of Cardiac Surgery, Universitätsklinikum Magdeburg, Magdeburg 39120 , Germany; Department of Cardiac Surgery, Herzzentrum Dresden Universitatsklinik, Dresden D-01307, Germany; Department of Cardiac Surgery, Klinikum Oldenburg GGMBH AoR, Oldenburg 26133, Germany; Department of Cardiac Surgery, Universitätsklinikum Essen, Essen 45122, Germany; Department of Cardiac Surgery, Universitätsklinikum Augsburg, Augsburg 86156, Germany; Department of Cardiac Surgery, Fondazione Poliambulanza Istituto Ospedaliero, Brescia 25124, Italy; Department of Cardiac Surgery, Ospedale del Cuore di Massa, Massa 54100, Italy; Department of Cardiac Surgery, Ospedale Ca’ Foncello di Treviso, Treviso 31100, Italy; Department of Cardiac Surgery, Az. Ospedaliero-Universitaria “Ospedali Riuniti” di Trieste, Trieste 34149, Italy; Department of Cardiac Surgery, A.O.U. Città della Salute e della Scienza di Torino—Ospedale Molinette, Torino 10126, Italy; Department of Cardiac Surgery, Policlinico S.Orsola-Malpighi, Bologna 40138, Italy; Department of Cardiac Surgery, Policlinico Paolo Giaccone, Palermo 90127, Italy; Department of Cardiac Surgery, Azienda Ospedaliera dei Colli—Ospedale Monaldi, Napoli 80131, Italy; Department of Cardiac Surgery, Catharina Ziekenhuis, Eindhoven 5623 EJ, The Netherlands; Department of Cardiac Surgery, Dedinje Cardiovascular Institute, Belgrade 11040, Serbia; Department of Cardiac Surgery, Inselspital, Universitätsspital Bern, Bern 3010, Switzerland; Department of Cardiac Surgery, King’s College Hospital, London SE5 9RS, United Kingdom; Department of Cardiac Surgery, Yonsei University Severance Cardiovascular Hospital, Seoul 03722, South Korea; Department of Cardiac Surgery, Seoul National University Hospital, Seoul 03722, South Korea; Department of Cardiac Surgery, Pusan National University Yangsan Hospital, Pusan 50612, South Korea; Department of Cardiac Surgery, Hospital de Santa Maria Lisbon, Lisbon 1649-035, Portugal

**Keywords:** sutureless aortic valve, Perceval Plus, sex differences, minimally invasive surgery, clinical outcomes, meta-regression

## Abstract

**Objectives:**

Sutureless aortic valve prostheses reduce surgical times and facilitate minimally invasive approaches, improving patient outcomes. However, it remains unclear whether these devices provide specific benefits to female patients, in whom sex-related differences in valve surgery outcomes remain a matter of debate.

**Methods:**

Up to September 2024, 535 subjects (261 women) received Perceval Plus at 35 investigational sites from Mitral, Aortic aNd Tricuspid Post-maRket Study in a reAl-world Setting observational prospective registry. Moreover, meta-regression was performed to assess whether sex modifies outcomes.

**Results:**

Men have a larger body size (body surface area: male 2.0 ± 0.2 vs female 1.8 ± 0.2, *P* < .001), resulting in a larger prosthesis size (size S: male 2.9% vs female 34.9%). Surgery was still significantly faster in women (cross-clamp time: male 63.7 ± 29.4 min vs female 56.8 ± 29.4 min, *P* = .002), partly because approximately 10% of procedures in male patients were combined, increasing duration. Early outcomes were comparable between sexes (hospital deaths: male 3 [1.1%] vs female 5 [1.9%], *P* = .49). At follow-up, no significant differences were observed (follow-up deaths: male 9 [3.3%] vs female 5 [1.9%], *P* = .42). Meta-regression showed no effect of female sex prevalence.

**Conclusions:**

Our registry and meta-regression analysis did not reveal significant differences in outcomes between men and women. Preoperative characteristics, however, differ between sexes and may influence outcomes and prosthesis choice. Long-term conclusions are limited by the current follow-up duration and will be further explored as data collection progresses.

**Clinical registration number:**

NCT05002543, ClinicalTrials.gov (https://clinicaltrials.gov/study/NCT05002543)

## Graphical abstract

**Figure:**
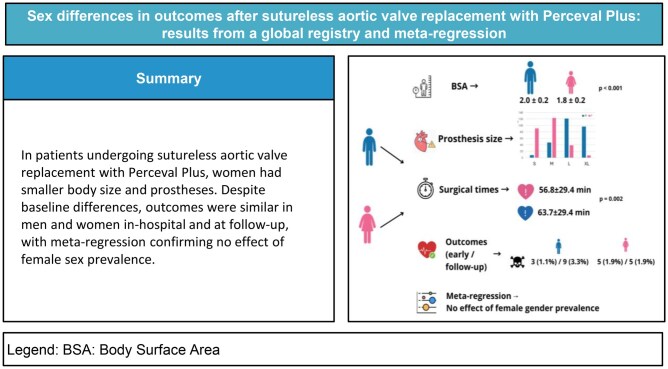

## INTRODUCTION

Female sex has historically been included as an independent risk factor in commonly used cardiac surgical risk scores, such as EuroSCORE II and the STS score.^1^^,^^2^ However, the interpretation of sex-related risk has been challenged by the historically low representation of women in cardiovascular clinical trials, which has limited the robustness of evidence specifically addressing female patients and the generalizability of study findings.^3^

Furthermore, it is well documented that women are often referred to cardiac surgery later than men.^4^ As a consequence, especially in valve surgery, earlier observational studies reported higher mortality and selected complication rates in female patients.^5^^,^^6^ However, these data largely derive from older series and more recent analyses based on larger cohorts suggest increasingly comparable outcomes between sexes when contemporary surgical strategies are applied.^7–9^

In this context, minimally invasive access does not consistently appear to offset outcome differences,^10^ despite minimally invasive surgery having generally been shown to reduce hospital mortality and improve outcomes for patients with predicted mortality scores.^11^

Sutureless aortic valve prostheses fit into this context, with evidence suggesting a potential reduction in postoperative complications compared with conventional prostheses,^12^ probably thanks to their ability to significantly reduce ischemic times of the intervention by about a third.^13^ However, this constantly expanding technology offers several advantages, especially when combined with minimally invasive approaches. Nevertheless, patient characteristics may influence these improved outcomes,^14^ and it is currently unknown whether these prostheses pose a lower risk to female patients compared with male patients.

Although the sex effect has been well characterized in some contexts, such as transcatheter and conventional aortic valve surgery,^15^ a significant knowledge gap still exists regarding patients undergoing sutureless aortic valve replacement.

This study aims to analyse data extracted from a global registry of cardiac valve prostheses, including hospital-based data collection and patient follow-up, to compare outcomes after sutureless aortic valve prosthesis implantation between male and female patients.

## METHODS

### Study design and patient enrollment

Our data were extracted from MANTRA (Mitral, Aortic aNd Tricuspid post-maRket study in a reAl-world setting), an observational and prospective study sponsored by Corcym to evaluate products used in a real-world setting in the post-marketing phase. Details on the study design and end-points have been previously published.^16^

Collected data included demographics, medical history, procedural details, in-hospital monitoring, and follow-up. Sex was recorded as reported in the patients’ medical records at each participating site. Gender identity was not specifically collected.

All patients eligible for valve treatment with any commercially available Corcym heart device were offered to participate in the study. Patients were enrolled prospectively.

The study was registered on *ClinicalTrials.gov* (Clinical registration number: NCT05002543) and was conducted in accordance with the latest version of the Declaration of Helsinki, Good Clinical Practices defined in ISO 14155:2020, data protection laws, and pertinent individual country laws and regulations.

Appropriate submissions to relevant Ethics Committees and/or health authorities were performed according to local regulations.

All subjects enrolled in the trial provided written informed consent, approved by the EC/IRB at each participating centre. All pre-, intra-, postoperative, and follow-up parameters were collected and compared for male and female patients.

The study was conducted using a risk-based monitoring approach that included a combination of on-site monitoring and centralized monitoring (evaluation without visiting the clinical study site), including full or partial source data verification. Data completeness and accuracy were reviewed, and discrepancies were resolved through queries before database update.

As part of the study, subjects completed quality of life questionnaires at baseline, 30 days, and at 1-year follow-up. The Kansas City Cardiomyopathy Questionnaire (KCCQ) scores range from 0 to 100 and EQ-5D values are reported using the EQ-VAS format, which also ranges from 0 to 100.

From December 2019 to September 2024, 535 subjects were enrolled and implanted with Perceval Plus at 35 investigational sites (**Appendix 1**). Enrollment and data collection are ongoing. At the time of the database snapshot for this analysis, several patients still had open follow-up visit windows based on their individual inclusion dates. The 30-day visit was completed for 454 subjects (88.0%), while the 1-year visit was completed for 282 subjects (77.5%). Later follow-up availability was limited by the enrollment timeline, with only one patient reaching 5 years (**Table 1**).

**Table 1.** Subject Disposition and Compliance Follow-up

Visit interval	Expected (%)	Visit performed (%)	Visit not performed (%)	Visit data pending entry (%)
Preoperative	535 (100%)	535 (100%)	–	–
Discharge	529 (100%)	526 (99.4%)	–	3 (0.6%)
30 Days	516 (100%)	454 (88.0%)	6 (1.2%)	56 (10.9%)
12 Month	364 (100%)	282 (77.5%)	7 (1.9%)	75 (20.6%)
2 Year	120 (100%)	32 (26.7%)	1 (0.8%)	87 (72.5%)
3 Year	45 (100%)	32 (71.1%)	–	13 (28.9%)
4 Year	43 (100%)	31 (72.1%)	–	12 (27.9%)
5 Year	7 (100%)	1 (14.3%)	–	6 (85.7%)
6 Year	4 (100%)	–	–	4 (100%)

### Meta-regression analysis

In addition to study data, a meta-regression was performed to assess potential sex effects in the literature. The analysis followed the Preferred Reporting Items for Systematic Reviews and Meta-Analyses (PRISMA) statement,^17^ and the research strategy was developed according to the recommendations of the Cochrane Collaboration.

Following the PICO framework:

- Population (P): adult patients (>18 years) undergoing sutureless aortic valve replacement;
- Intervention (I): use of sutureless aortic valve prostheses (Perceval or equivalent);
- Comparison (C): clinical outcomes according to sex (female vs male);
- Outcomes (O): mortality outcomes, including hospital mortality as the main outcome, and follow-up mortality and overall survival as secondary outcomes, when reported by the included studies. Length of follow-up was extracted to contextualize survival and follow-up mortality estimates.

A broad, computerized literature search was performed to identify all relevant studies from the PubMed database exploring sex-related Medical Subject Heading (MeSH) terms in the “sutureless aortic valve” population. The PubMed database was searched by entering the following key words: (“sutureless aortic valve” [Mesh] OR “sutureless aortic valve replacement”) AND “gender” [Mesh]. We restricted the research to English publications. The database was last accessed on July 1, 2025. The search was limited to studies conducted on human recipients. Study selection is shown in the PRISMA flow diagram (**Appendix 5**).

Studies were included in the final analysis if: patients were >18 years (I), >100 patients were included in the main analysis, in order to provide the most consistent interpretation of the clinical series data (II), studies provided a description of sex of the population (III). Furthermore, articles without the possibility of extracting the presence of men and women were excluded. Abstracts, case reports, conference presentations, editorials, reviews, and expert opinions were excluded. Systematic reviews, meta-analyses, and non-relevant cardiac surgery studies were also excluded. Selected articles underwent extensive evaluation at title and abstracts level by 2 independent researchers (G.S. and M.D.M.) to assess the potential inclusion in the meta-analysis. Discrepancies were solved by consensus with the intervention of a third reviewer (R.L.). There were no duplicate data.

### Statistics

Continuous variables were summarized using mean, standard deviation (SD), median, first and third quartiles (Q1 and Q3), minimum, and maximum values. Categorical variables were presented as frequency counts and percentages.

For the purposes of this analysis, missing data were handled through listwise deletion: patients with incomplete data for a given variable were excluded from the corresponding analysis of that variable. No imputation was performed.

The normality of continuous variables was assessed using the Shapiro-Wilk test. If the normality assumption was met, the independent samples t-test was employed; otherwise, the Mann-Whitney U test was utilized.

Fisher’s exact test was performed to compare categorical variables.

The proportions of early (defined as occurring up to 30 days) and late (>30 days) adverse events were calculated as the total number of events divided by the total number of patients. Linearized complication rates (and 2-sided 95% confidence intervals [CIs]) were calculated as the number of late events (>30 days) divided by the number of late patient-years.

Adjusted least-squares means of change from baseline were estimated using a linear mixed-effects model. The model included baseline value, time (as a categorical variable), sex (male vs female), and the interaction between time and sex as fixed effects, with subject as a random effect. At each post-baseline time point (hospital discharge, 30 days, 1 year, 2 years, 3 years, and 4 years), LS means for males and females were computed, as well as the differences (male minus female), along with 95% CIs and *P-*value. A *P-*value of less than 0.05 was considered statistically significant. Missing follow-up data were handled under the Missing At Random (MAR) assumption, as missingness was primarily attributable to nonattendance or pending visits and was balanced between sexes. Mixed-effects models allow valid inference under MAR; therefore, no multiple imputation or additional missing-data sensitivity analyses were performed.

A sensitivity analysis was performed using adjusted mixed-effects models including clinically relevant covariates (age, coronary artery disease, pulmonary hypertension, smoking status, surgical approach, concomitant procedures, and CABG) (see **Appendix 8**).

Concerning the meta-regression analysis, the overall proportion of female patients and events was calculated from studies that reported a single proportion using a meta-analytic approach, employing the *metaprop* function of the *meta package* in R. A logit-transformation was performed to calculate CIs for individual study results; Clopper-Pearson approach was used, and DerSimonian-Laird estimator was used to estimate the variance between studies. The total proportion with 95% CI was reported. Funnel plot and Egger’s test were used to estimate publication bias. Risk of bias for the studies included in the systematic review was assessed using the Newcastle-Ottawa Scale (NOS) (see **Appendix 4**).

Meta-regression was then applied to verify the influence of female prevalence in each study on pooled proportion of clinical events, referred to deaths reported as hospital mortality and/or follow-up mortality, according to data availability in the included studies. A mixed-effects meta-regression model, the predicted average prevalence of events as a function of the predictor (gender prevalence), was used. The mixed-effects model allows for within-study variation and between-study variation and is therefore taken as the most flexible model to choose in many applications. In our case, female prevalence is fixed effect and intercept is random effect. Residual heterogeneity was assessed using the test for residual heterogeneity (QE), and the *H*^2^ value was reported.

“*Meta, Metafor and Metaprop packages*” in R-studio version 2025.05.0 + 496 (2025) were used.

## RESULTS

**Table 2** presents the pre- and intraoperative data of the patients included in the study. Male subjects have a larger body size, resulting in larger implanted prostheses. Women present for surgery with a higher predicted risk, according to the EuroSCORE II and STS measurements^4^ (**Table 2**). Postoperative characteristics and early (≤30 days) outcomes are reported in **Table 3**.

**Table 2.** Pre- and Intraoperative Characteristics

	Male *N* = 274	Female *N* = 261	Total *N* = 535	*P*
Age, years Median (IQR)	73 (68-78)	73 (69-77)	73 (68-77)	.43
Height, cm Mean ± SD	172.6 ± 7.5	159.7 ± 7.3	166.5 ± 9.9	<.001
Weight, kg Median (IQR)	82.0 (73.0-94.0)	72.0 (62.3-84.0)	78.0 (67.0-88.9)	<.001
BMI, kg/m^2^ Median (IQR)	27.4 (24.9-31.1)	28.2 (24.2-32.3)	27.7 (24.7-31.6)	.51
BSA, m^2^ Median (IQR)	2.0 (1.9-2.1)	1.8 (1.7-1.9)	1.9 (1.7-2.0)	<.001
EuroSCORE II Median (IQR)	1.8 (1.1-3.3)	2.0 (1.3-4.0)	1.9 (1.7-2.0)	.020
STS score Median (IQR)	1.6 (1.1-3.1)	2.7 (1.8-4.5)	2.1 (1.3-3.9)	<.001
Redo, *N* (%)	69 (27.4%)	48 (20.3%)	117 (24.0%)	.070
Cerebrovascular accident, *N* (%)	18 (7.8%)	12 (5.4%)	30 (5.6%)	.35
Conduction disorder, *N* (%)	27 (11.7%)	15 (7.0%)	42 (7.9%)	.105
Atrial fibrillation, *N* (%)	26 (11.5%)	25 (11.5%)	51 (9.5%)	>.999
Coronary artery disease, *N* (%)	144 (56.0%)	89 (36.6%)	233 (46.6%)	<.001
Diabetes mellitus, *N* (%)	89 (34.6%)	94 (39.0%)	183 (36.7%)	.35
Pulmonary hypertension, *N* (%)	29 (11.9%)	45 (19.1%)	74 (15.4%)	.032
Tobacco user, *N* (%)	121 (50.8%)	55 (23.6%)	176 (37.4%)	<.001
Surgical approach, *N* (%)				
Median sternotomy	178 (65.0%)	144 (55.2%)	322 (60.2%)	.022
Minimally invasive	96 (35.0%)	117 (44.8%)	213 (39.8%)	
Mini-sternotomy	49 (51%)	62 (53%)	111 (52.1%)	
Mini-thoracotomy	47 (49%)	55 (47%)	102 (47.9%)	
Bicuspid, N (%)	42 (16.7%)	32 (13.3%)	74 (15.0%)	.32
Perceval Plus size, N (%)				
S	8 (2.9%)	91 (34.9%)	99 (18.5%)	<.001
M	47 (17.2%)	124 (47.5%)	171 (32.0%)	
L	122 (44.5%)	39 (14.9%)	161 (30.1%)	
XL	97 (35.4%)	7 (2.7%)	104 (19.4%)	
Concomitant procedures, N (%)	123 (49.0%)	92 (38.2%)	215 (43.7%)	.018
CABG	102 (37.2%)	53 (20.3%)	155 (29.0%)	<.001
Mitral valve repair/replacement	11 (4%)	12 (4.6%)	23 (4.3%)	.83
Tricuspid valve repair/replacement	5 (1.8%)	5 (1.9%)	10 (1.9%)	>.999
Pulse generator implant	0	1 (0.4%)	1 (0.2%)	.49
AF treatment	23 (8.4%)	22 (8.4%)	45 (8.4%)	>.999
Septal myectomy	1 (0.4%)	2 (0.8%)	3 (0.6%)	.62
Atrial septal defect repair	0	2 (0.8%)	2 (0.4%)	.24
Ventricular septal defect repair	0	1 (0.4%)	1 (0.2%)	.49
Aortic root replacement	3 (1.1%)	6 (2.3%)	9 (1.7%)	.33
Aortic root enlargement	0	0	0	NA
Other procedures	16 (5.8%)	16 (6.1%)	32 (6.0%)	>.999

Abbreviations: AF, atrial fibrillation; BMI, body mass index; BSA, body surface area; CABG, coronary artery bypass grafting; IQR, interquartile range.

**Table 3.** Postoperative Characteristics, Early (≤30 Days) Outcomes

	Male *N* = 274	Female *N* = 261	Total *N* = 535	*P*
Skin-to-skin time, min, median (IQR)	194.0 (151.0-250.0)	175.0 (127.0-235.0)	185.0 (138.0-242.0)	.002
Cardio-pulmonary bypass time, min, median (IQR)	85.0 (66.0-118.0)	76.0 (53.0-105.0)	81.0 (59.0-113.0)	.003
Cross-clamp time, min, median (IQR)	56.5 (43.0-81.5)	49.0 (37.0-68.0)	53.0 (40.0-75.0)	.002
ICU ventilation time, hours, median (IQR)	9.0 (6.0-12.0)	9.0 (6.0-13.0)	9.0 (6.0-12.0)	.37
ICU duration, days, median (IQR)	2.0 (1.0-3.0)	2.0 (1.0-4.0)	2.0 (1.0-4.0)	.68
Duration of hospitalization, days, median (IQR)	9.0 (7.0-13.0)	10.0 (8.0-15.0)	10.0 (7.0-13.0)	.26
All deaths, *N* (%)	3 (1.1%)	5 (1.9%)	8 (1.5%)	.49
Stroke, *N* (%)	2 (0.7%)	4 (1.5%)	6 (1.1%)	.68
TIA, *N* (%)	0	0	0	NA
Endocarditis, *N* (%)	0	0	0	NA
Reintervention, *N* (%)	0	0	0	NA
Myocardial infarction, *N* (%)	1 (0.4%)	0	1 (0.2%)	>.999
Atrial fibrillation, *N* (%)	11 (4.0%)	9 (3.4%)	20 (3.7%)	.64
Acute kidney injury, *N* (%)	7 (2.6%)	6 (2.3%)	13 (2.4%)	>.999
Pacemaker implants, *N* (%)	14 (5.1%)	6 (2.3%)	20 (3.7%)	.112

Abbreviations: ICU, intensive care unit; TIA, transient ischemic attack.

At follow-up, both male and female patients showed sustained improvements in dyspnoea (NYHA class) and quality-of-life measures, with no significant differences, including EQ-5D and KCCQ scores at 12 months (EQ-5D *P* = .33; KCCQ *P* = .106) (**Table 4**).

**Table 4.** Follow-up Results

	Male		Female		Overall		
LATE events >30 days	*N* = 274	pt-yrs = 221.489 lin./rate 95% CI	*N* = 261	pt-yrs = 234.092 lin./rate 95% CI	*N* = 535	pt-yrs = 455.581 lin./rate 95% CI	*P*
Atrial fibrillation, *N* (%)	1 (0.4%)	0.5 (0.0-1.4)	0	0.0 (0.0-0.0)	1 (0.2%)	0.2 (0.0-0.7)	>.999
Stroke, *N* (%)	2 (0.7%)	0.9 (0.0-2.3)	2 (0.8%)	0.9 (0.0-2.1)	4 (0.7%)	0.9 (0.2-1.8)	>.999
Endocarditis, *N* (%)	2 (0.7%)	0.9 (0.0-2.3)	1 (0.4%)	0.4 (0.0-1.3)	3 (0.6%)	0.7 (0.0-1.5)	>.999
TIA, *N* (%)	1 (0.4%)	0.5 (0.0-1.4)	1 (0.4%)	0.4 (0.0-1.3)	2 (0.4%)	0.4 (0.0-1.1)	>.999
All deaths, *N* (%)	9 (3.3%)	4.1 (1.8-6.8)	5 (1.9%)	2.1 (0.4-4.3)	14 (2.6%)	3.1 (1.5-4.8)	.42
All reinterventions, *N* (%)	3 (1.1%)	1.4 (0.0-3.2)	0	0.0 (0.0-0.0)	3 (0.6%)	0.7 (0.0-1.5)	.25
Reinterventions due to endocarditis, *N* (%)	3 (1.1%)	1.4 (0.0-3.2)	0	0.0 (0.0-0.0)	3 (0.6%)	0.7 (0.0-1.5)	.25
Pacemaker implants, *N* (%)	2 (0.7%)	0.9 (0.0-2.3)	1 (0.4%)	0.4 (0.0-1.3)	3 (0.6%)	0.7 (0.0-1.5)	1.00

	Male *N* = 274		Female *N *= 261		Total *N* = 535		*P*
NYHA							
Preoperative (missing 0-2), mean ± SD	2.6 ± 0.7		2.4 ± 0.7		2.5 ± 0.1		.015
Discharge (missing 81-113), mean ± SD	2.4 ± 0.8		2.1 ± 0.8		2.3 ± 0.2		.45
30 days (missing 48-67), mean ± SD	1.4 ± 0.6		1.6 ± 0.6		1.5 ± 0.6		.32
12 months (missing 146-126), mean ± SD	1.3 ± 0.6		1.4 ± 0.6		1.3 ± 0.6		.32
24 months (missing 260-247), mean ± SD	1.4 ± 0.6		1.4 ± 0.6		1.4 ± 0.6		.84
36 months (missing 260-243), mean ± SD	1.4 ± 0.5		1.6 ± 0.7		1.5 ± 0.6		.86
48 months (missing 260-244), mean ± SD	1.4 ± 0.5		1.5 ± 0.5		1.4 ± 0.5		.94
EQ-5D (VAS)							
Preoperative (missing 26-30), mean ± SD	66.3 ± 20.1		62.2 ± 20.7		64.4 ± 20.5		.028
30 days (missing 60-62), mean ± SD	73.0 ± 17.2		70.8 ± 18.4		71.9 ± 17.8		.39
12 months (missing 154-138), mean ± SD	81.0 ± 11.8		77.3 ± 14.9		79.1 ± 13.5		.33
KCCQ							
Preoperative (missing 26-30), mean ± SD	61.2 ± 23.1		54.3 ± 22.6		57.9 ± 23.1		.001
30 days (missing 56-62), mean ± SD	74.4 ± 21.4		67.6 ± 22.5		71.2 ± 22.2		.008
12 months (missing 153-137), mean ± SD	88.1 ± 14.5		81.9 ± 18.8		85.0 ± 17.1		.106

*P*-values for NYHA, EQ-5D, and KCCQ were derived from mixed-effects models for repeated measures, assessing longitudinal differences between male and female patients.

Abbreviations: EQ-5D (VAS), EuroQol 5-dimensions questionnaire (visual analogue scale); KCCQ, Kansas City Cardiomyopathy Questionnaire; pt-yrs, patient-years.

Specifically, NYHA class significantly improved from baseline in both sexes during early follow-up and remained lower over time, with no significant sex-related differences at any assessed time point.

At 30 days, both sexes experienced pronounced declines compared to baseline (males: LS Mean estimate = −1.1543, *P* < .0001; females: LS Mean estimate = −0.9207, *P* < .0001). These improvements persisted at the 12-month assessment (males: −1.4216, *P* < .0001; females: −1.1256, *P* < .0001), indicating a continued improvement in functional status. At 2 years, the reduction remained significant for females (−1.3089, *P* = .043), while in males, the change was not statistically significant (*P* = .083). By the 3-year follow-up, neither group showed a statistically significant difference relative to baseline. These findings suggest a sustained trajectory of NYHA improvement over time. No statistically significant differences in LS Means differences were detected when comparing males and females at any assessed time point (see **Appendix 6**).

At the echocardiographic level, early postoperative gradients were slightly higher in female patients. This finding is consistent with the smaller prosthesis sizes implanted in women, reflecting their lower body surface area. This may have resulted in a transient, mild patient-prosthesis mismatch. However, this difference was no longer observed at follow-up, as gradients progressively normalized and remained comparable between sexes (**Table 5**), despite the limitations related to incomplete echocardiographic follow-up.

**Table 5.** Echocardiographic Results

	Male *N* = 274	Female *N* = 261	Total *N* = 535	*P*
MPG, mmHg, mean ± SD				
Preoperative (missing 31-30)	43.5 ± 17.6	47.4 ± 18.2	45.4 ± 18.0	.010
Discharge (missing 67-60)	10.0 ± 4.9	12.6 ± 5.2	11.3 ± 5.2	<.001
30 days (missing 77-105)	9.4 ± 4.2	11.0 ± 4.2	10.1 ± 4.3	.007
12 months (missing 163-148)	9.9 ± 4.6	12.2 ± 5.2	11.1 ± 5.0	<.001
24 months (missing 268-256)	9.9 ± 3.4	10.8 ± 3.8	10.3 ± 3.4	.68
36 months (missing 264-249)	10.0 ± 3.9	9.3 ± 3.6	9.6 ± 3.7	.91
48 months (missing 268-255)	9.7 ± 3.6	10.7 ± 4.6	10.2 ± 4.0	.42
PPG, mmHg, mean ± SD				
Preoperative (missing 55-45)	71.3 ± 26.8	77.6 ± 29.7	74.4 ± 28.4	.027
Discharge (missing 73-74)	18.8 ± 8.7	23.1 ± 8.7	20.9 ± 8.9	<.001
30 days (missing 79-106)	17.8 ± 9.4	19.8 ± 7.3	18.7 ± 8.6	.23
12 months (missing 162-152)	18.1 ± 7.3	21.4 ± 8.3	19.7 ± 7.9	.011
24 months (missing 269-257)	18.2 ± 6.3	16.0 ± 2.2	17.2 ± 4.8	.88
36 months (missing 264-249)	19.8 ± 6.6	18.1 ± 7.2	18.9 ± 6.8	.73
48 months (missing 268-255)	17.6 ± 5.3	19.3 ± 8.1	18.5 ± 6.6	.86
EOA, cm^2^, mean ± SD				
Preoperative (missing 128-125)	0.8 ± 0.3	0.8 ± 0.6	0.8 ± 0.5	.036
Discharge (missing 228-210)	1.9 ± 0.6	1.5 ± 0.4	1.7 ± 0.5	.010
30 days (missing 194-203)	1.8 ± 0.6	1.6 ± 0.6	1.7 ± 0.6	.112
12 months (missing 226-225)	1.9 ± 0.6	1.4 ± 0.4	1.7 ± 0.6	<.001
24 months (missing 271-259)	2.4 ± 0.9	1.9 ± 0.1	2.2 ± 0.7	.72
36 months (missing 271-254)	1.9 ± 0.4	1.6 ± 0.4	1.7 ± 0.4	.81
48 months (missing 273-259)	2.3	1.5 ± 0.2	1.8 ± 0.5	NA
LV mass, g, mean ± SD				
Preoperative (missing 189-190)	227.4 ± 64.6	188.8 ± 57.2	209.8 ± 64.1	.001
Discharge (missing 217-207)	201.5 ± 63.2	170.6 ± 68.0	186.5 ± 69.8	>.999
30 days (missing 199-201)	194.1 ± 63.1	164.2 ± 74.7	180.8 ± 69.8	.30
12 months (missing 229-229)	202.8 ± 66.2	146.4 ± 49.8	179.3 ± 65.8	.190
24 months (missing 272-259)	163.4 ± 94.2	131.6 ± 37.6	147.5 ± 61.4	.97
36 months (missing 270-253)	231.2 ± 88.7	188.8 ± 78.5	202.9 ± 80.7	NA
48 months (missing 268-256)	196.2 ± 50.2	183.4 ± 41.8	190.4 ± 44.7	NA
LVEF, %, mean ± SD				
Preoperative (missing 30-26)	55.6 ± 10.6	58.4 ± 8.9	56.9 ± 9.9	.001
Discharge (missing 81-68)	55.2 ± 9.3	58.2 ± 7.7	56.6 ± 8.7	NA
30 days (missing 80-102)	55.7 ± 7.8	58.1 ± 6.8	56.9 ± 7.4	NA
12 months (missing 155-138)	57.9 ± 6.7	58.7 ± 7.0	58.3 ± 6.9	NA
24 months (missing 268-256)	55.0 ± 16.1	57.0 ± 7.0	56.0 ± 12.5	NA
36 months (missing 263-247)	60.5 ± 9.7	64.1 ± 12.2	62.3 ± 11.1	NA
48 months (missing 268-255)	62.2 ± 5.4	65.0 ± 5.5	63.6 ± 5.6	NA
LVEDD, mm, mean ± SD				
Preoperative (missing 141-108)	49.0 ± 7.2	45.6 ± 7.6	47.3 ± 7.6	.003
LVESD, mm, mean ± SD				
Preoperative (missing 172-138)	31.4 ± 7.6	29.3 ± 7.1	30.4 ± 7.4	.047

*P*-values for MPG, PPG, EOA, and LV mass were derived from mixed-effects models for repeated measures, assessing longitudinal differences between male and female patients. *P*-values for LVEF, LVEDD, and LVESD were calculated at baseline using the t-test or Mann-Whitney U test, as appropriate.

Abbreviations: EOA, effective orifice area; LV mass, left ventricular mass; LVEDD, left ventricular end-diastolic diameter; LVEF, left ventricular ejection fraction; LVESD, left ventricular end-systolic diameter; MPG, mean pressure gradient; PPG, peak pressure gradient.

To account for baseline and procedural differences between sexes, adjusted analyses were performed. In these models, CABG was not significantly associated with any of the investigated outcomes and findings remained consistent with the primary unadjusted analysis (see **Appendix 8**).

**Figure 1.**
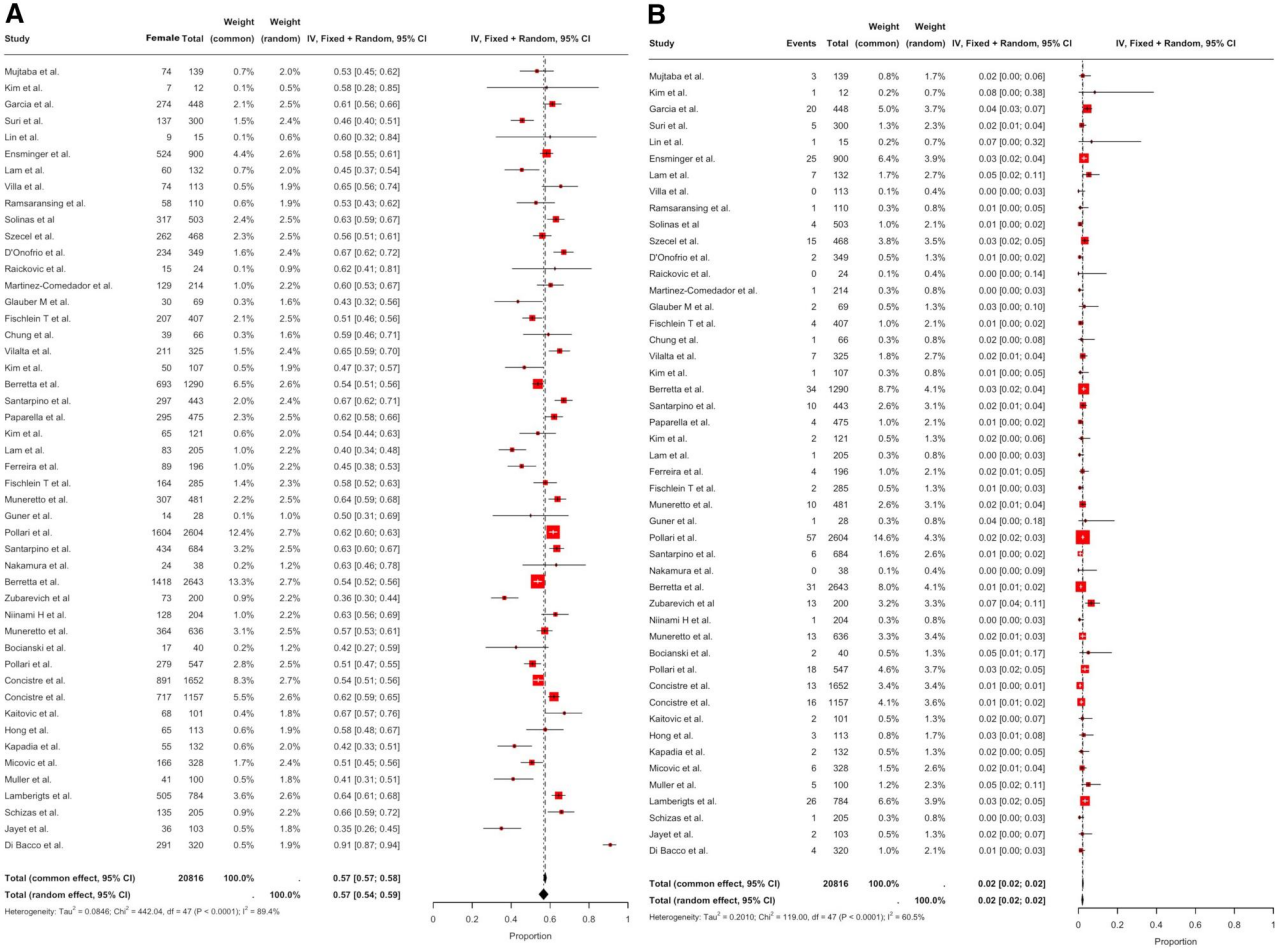
Forest Plots of Pooled Study-level Proportions. (A) Forest plot showing the study-level prevalence of female patients across 48 included cohorts. (B) Forest plot showing the study-level prevalence of mortality events across the same studies. Random-effects model with logit transformation

**Figure 2.**
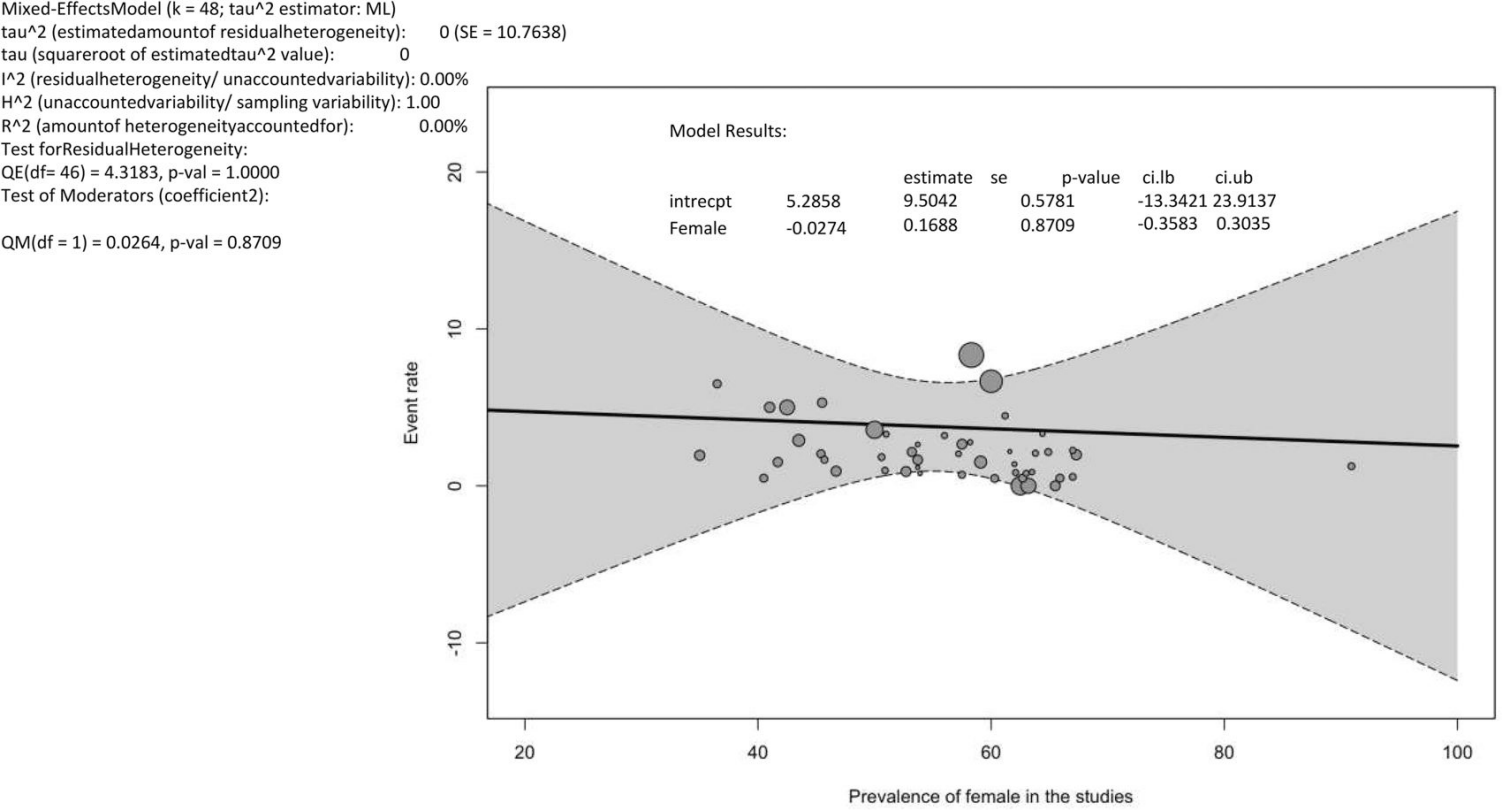
Mixed-Effects Meta-Regression Evaluating the Association Between the Proportion of Female Patients (*x*-Axis) and the Study-Level Mortality Event Rate (*y*-Axis). Bubble size is proportional to study weight. No significant association was found (*P* = .8709)

The literature review yielded 48 articles on the topic, including articles that highlighted the percentages of men and women in their case studies (see **Appendix 2**). The characteristics of the analysed studies, particularly the percentages by sex, are shown in **Appendix 3**. The pooled prevalence of the female sex on 20 816 patients was 57% [54%-59%] (**Figure 1A**); the pooled prevalence of mortality events was 2% [2%-2%] (**Figure 1B**). The prevalence of female sex in each study showed no impact on the pooled prevalence of the mortality events (**Figure 2**). The mixed-effects meta-regression demonstrated no residual heterogeneity (τ^2^ = 0; *H*^2^ = 1.00; *I*^2^ = 0%). The test for residual heterogeneity was nonsignificant (QE = 4.3183, df = 46, *P* > .999), confirming perfect homogeneity among studies. The moderator (female prevalence) was not associated with mortality event rates (estimate −0.0274, SE 0.1688, *P* = .87). In other words, whether it was underrepresented or overrepresented, the results were similar.

## DISCUSSION

The results of our study demonstrate that male and female patients receiving Perceval Plus have different baseline characteristics. On average, males present with a greater association with ischemic heart disease, which explains the higher prevalence of combined procedures in this patient group, a longer duration of aortic clamping, and a higher percentage of patients who undergo complete sternotomy rather than minimally invasive surgery. It should also be noted that the significantly higher prevalence of coronary artery disease in male patients often requires concomitant coronary artery bypass grafting. This circumstance increases operative complexity and may contribute to outcome differences between sexes. In contrast, female patients more frequently presented with pulmonary hypertension, which may have influenced risk scores and perioperative management in a different manner. Among female patients, the higher percentage of isolated pathology allows for a higher percentage of patients eligible for a minimally invasive procedure, and their smaller body size explains the smaller average prosthesis size. Additionally, anatomical characteristics typically observed in female patients—such as smaller annular dimensions and the lower prevalence of coronary artery disease requiring concomitant revascularization—make them more suitable for minimally invasive procedures that are facilitated by sutureless prostheses, thereby contributing to their higher adoption in women. Therefore, the comparison between these 2 groups inherently carries an implicit statistical bias, reflecting a classic apples-to-oranges comparison.

Indirectly, these findings suggest that female patients, despite a higher predicted operative risk and a greater proportion of isolated and minimally invasive procedures, achieved postoperative outcomes comparable to those of men, who more frequently underwent combined surgery via full sternotomy, a factor that also increases operative risk. This pattern may reflect the coexistence of a higher baseline risk profile in women and a higher procedural burden in men, within different clinical and surgical pathways.

Importantly, EuroSCORE II and STS risk predictive scores indicate a higher average predicted mortality in female patients. In this context, the use of sutureless prostheses within minimally invasive approaches may contribute to procedural efficiency, potentially facilitating comparable outcomes across groups, although no definitive conclusions regarding sex-specific effects can be drawn from the present data.

Consistent with this interpretation, available literature on sutureless aortic valve replacement has not demonstrated a clear or consistent male/female sex effect, with cases in which women were more frequently subjected to the sutureless procedure and other cases in which no such female prevalence was found.

Given the limited number of adverse events and the relatively short duration of follow-up currently available, the present findings reflect interim observations from a real-world registry, with longer follow-up needed to further clarify potential sex-related effects.

Notably, the use of the Perceval sutureless valve, allowing for a larger size implantation due to its “stentless” design (absence of the sewing ring), is particularly indicated for small women for whom otherwise a higher risk of mismatch may occur.^18^ In line with this concept, the prevalence of patients requiring aortic root enlargement in our cohort was zero, as the Perceval valve’s stentless-like design minimizes the risk of patient-prosthesis mismatch, thereby reducing the need for enlargement procedures. Given that echocardiographic follow-up was available only for a subset of patients, we observed slightly higher early postoperative gradients in women, likely reflecting the smaller prosthesis sizes implanted. However, these differences were no longer present at follow-up, as gradients normalized and remained comparable between sexes. Dyspnoea and quality-of-life scores were comparable between women and men, despite the smaller average prosthesis size in women, considering the overall Sex × Visit interaction (Type III tests: NYHA *P* = .8271, EQ-5D *P* = .7850, KCCQ *P* = .6466).

Our meta-regression also showed that both in studies with higher and lower prevalence of female sex, the results were similar, proving the need for studies oriented towards this type of analysis by comparing populations of different sexes but with comparable preoperative characteristics.

### Limitations

A major limitation of this study is the incomplete long-term follow-up, with a limited number of patients beyond the 2-year mark, primarily due to the ongoing nature and progressive enrollment timeline of the MANTRA registry. Follow-up is ongoing and expected to continue over time, but conclusions regarding late outcomes should be interpreted with caution given the small sample size at longer time points. As the study continues, additional follow-up data will accumulate in accordance with the study schedule, enabling more robust long-term analyses in future updates.

A further limitation is that, although the study selection process for the meta-regression was designed to avoid any overlap, the inclusion of studies potentially related to MANTRA registry centres cannot completely exclude the risk of overlapping data. Regarding the literature synthesis, the meta-regression used a focused PubMed search restricted to English-language studies and MeSH terms related to “gender.” Although appropriate for the research aims, this approach may have omitted some relevant publications. Finally, although multivariable regression or propensity-matched analyses could further help isolate the independent contribution of sex, these approaches were not predefined in the present study. As the registry expands and follow-up becomes more complete, such adjusted analyses may be explored to provide additional insight.

## Supplementary Material

ivag170_Supplementary_Data

## Data Availability

The data underlying this article are available in the article and in its online supplementary material.

## ABBREVIATIONS

EOA: Effective orifice area
ICU: Intensive care unit
KCCQ: Kansas City Cardiomyopathy Questionnaire
LS Means: Least-squares means
MANTRA: Mitral, Aortic aNd Tricuspid post-maRket study in a reAl-world setting
MeSH: Medical Subject Headings
MPG: Mean pressure gradient
NYHA: New York Heart Association
PPG: Peak pressure gradient
PRISMA: Preferred Reporting Items for Systematic Reviews and Meta-Analyses
STS: Society of Thoracic Surgeons
