# Supplementary material for: Sex-Related Outcome After Sutureless Aortic Valve Replacement With Perceval Plus: Results From a Global Registry and Meta-Regression
Source: Interdiscip Cardiovasc Thorac Surg. 2026 Jun 3;41(6):ivag170. doi: 10.1093/icvts/ivag170 (PMC13278764; doi:10.1093/icvts/ivag170)
Supplement: ivag170_Supplementary_Data [file ivag170_supplementary_data.zip › Supplementary files - Appendix 2,3,4,5 (1).pdf]

Appendix 2. Studies included in the meta-regression

1) Zubarevich A, Amanov L, Arjomandi Rad A, Beltsios ET, Szczechowicz M, Osswald A, Ruhparwar A, Weymann A. Single-Center Real-World Experience with Sutureless Aortic Valve Prosthesis in Isolated and Combined Procedures. J Clin Med. 2023;12:4163.

2) Martınez-Comendador JM, Estevez-Cid F, Gonzalez Barbeito M, Velasco Garcıa De Sierra C, Bouzas Mosquera A, Barbeito C et al. Mid-term assessment of structural valve deterioration of perceval S sutureless prosthesis using the last European consensus definition. Interact CardioVasc Thorac Surg 2021;32:499–505.

3) Lam KY, Reardon MJ, Yakubov SJ, Modine T, Fremes S, Tonino PAL, Tan ME, Gleason TG, Harrison JK, Hughes GC, Oh JK, Head SJ, Huang J, Deeb GM. Surgical Sutureless and Sutured Aortic Valve Replacement in Low-risk Patients. Ann Thorac Surg. 2022;113:616-622.

4) Glauber M, Kent WDT, Asimakopoulos G, Troise G, Padrò JM, Royse A, Marnette JM, Noirhomme P, Baghai M, Lewis M, Di Bacco L, Solinas M, Miceli A. Sutureless Valve in Repeated Aortic Valve Replacement: Results from an International Prospective Registry. Innovations (Phila). 2021;16:273-279.

5) Kim DJ, Kim HH, Lee SY, Lee S, Chang BC. Early Clinical Experience with Sutureless Aortic Valve Replacement for Severe Aortic Stenosis. Korean J Thorac Cardiovasc Surg. 2018;51:1-7.

6) Fischlein T, Folliguet T, Meuris B, Shrestha ML, Roselli EE, McGlothlin A, Kappert U, Pfeiffer S, Corbi P, Lorusso R; Perceval Sutureless Implant Versus Standard-Aortic Valve Replacement Investigators. Sutureless versus conventional bioprostheses for aortic valve replacement in severe symptomatic aortic valve stenosis. J Thorac Cardiovasc Surg. 2021;161:920-932.

7) Niinami H, Sawa Y, Shimokawa T, Domoto S, Nakamura Y, Sakaguchi T, Ito T, Toda K, Amano A, Gersak B. 1-year outcomes of patients implanted with the Perceval sutureless valve: the Japanese post-marketing surveillance study. Heart Vessels. 2023;38:949-956.

8) Chung YH, Lee SH, Ko YG, Lee S, Shim CY, Ahn CM, Hong GR, Shim JK, Kwak YL, Hong MK. Transcatheter Aortic Valve Replacement versus Sutureless Aortic Valve Replacement: A Single Center Retrospective Cohort Study. Yonsei Med J. 2021;62:885-894.

9) Ferreira R, Rua N, Sena A, Velho TR, Gonçalves J, Junqueira N, Almeida AG, Nobre Â, Pinto F. Sutureless bioprosthesis for aortic valve replacement: Surgical and clinical outcomes. J Card Surg. 2022;37:4774-4782.

10) Lam KY, Akca F, Verberkmoes NJ, van Dijk C, Claessens A, Soliman Hamad MA, van Straten AHM. Conduction disorders and impact on survival after sutureless aortic valve replacement compared to conventional stented bioprostheses. Eur J Cardiothorac Surg. 2019;55:1168-1173.

11) Hong S, Son JW, Yoon Y. Clinical Midterm Results of Surgical Aortic Valve Replacement with Sutureless Valves. J Chest Surg. 2024;57:255-262.

12) Kapadia SJ, Salmasi MY, Zientara A, Roussin I, Quarto C, Asimakopoulos G. Perceval sutureless bioprosthesis versus Perimount sutured bioprosthesis for aortic valve replacement in patients with aortic stenosis: a retrospective, propensity-matched study. J Cardiothorac Surg. 2024;19:95.

13) Muneretto C, Di Bacco L, Di Eusanio M, Folliguet T, Rosati F, D'Alonzo M, Cugola D, Curello S, Palacios CM, Baudo M, Pollari F, Fischlein T. Sutureless and Rapid Deployment vs. Transcatheter Valves for Aortic Stenosis in Low-Risk Patients: Mid-Term Results. J Clin Med. 2023;12:4045.

14) Ramsaransing K, Hindori V, Kougioumtzoglou A, Kaya A, Verbeek E. Minimally Invasive Sutureless Aortic Valve Replacement With the Perceval S Bioprosthesis Through Ministernotomy: A Single-Center Experience. Cureus. 2020;12:e11212.

15) Micovic S, Nobre A, Choi JW, Solinas M, Shehada SE, Torella M, Baeza C, Parrino E, Pollari F, Troise G, Kappert U, Mellert F, Je HG, Argano V, Lam KY, Rinaldi M, Gutermann H, Meuris B; MANTRA Investigators. Early outcomes of aortic valve replacement with Perceval PLUS sutureless valve: results of the prospective multicentric MANTRA study. J Cardiothorac Surg. 2024;19:340.

16) Bociański M, Puślecki M, Olasińska-Wiśniewska A, Perek B, Stefaniak S, Buczkowski P, Jemielity M. A comparative study of minimally invasive aortic valve replacement with sutureless biological versus mechanical prostheses. Kardiochir Torakochirurgia Pol. 2023;20:77-82.

17) Pollari F, Mamdooh H, Hitzl W, Grossmann I, Vogt F, Fischlein T. Ten years' experience with the sutureless aortic valve replacement: incidence and predictors for survival and valve durability at follow-up. Eur J Cardiothorac Surg. 2023;63:ezac572.

18) Solinas M, Bianchi G, Chiaramonti F, Margaryan R, Kallushi E, Gasbarri T, Santarelli F, Murzi M, Farneti P, Leone A, Simeoni S, Varone E, Marchi F, Glauber M, Concistrè G. Right anterior mini-thoracotomy and sutureless valves: the perfect marriage. Ann Cardiothorac Surg. 2020;9:305-313.

19) Müller H, Szalkiewicz P, Benedikt P, Ratschiller T, Schachner B, Schröckenstein S, Zierer A.Single center real-world data and technical considerations from 100 consecutive patients treated with the Perceval aortic bioprosthesis. Front Cardiovasc Med. 2024;11:1417617.

20) Szecel D, Eurlings R, Rega F, Verbrugghe P, Meuris B. Perceval Sutureless Aortic Valve Implantation: Midterm Outcomes. Ann Thorac Surg. 2021;111:1331-1337.

21) Berastegui Garcı´a E, Camara Rosell ML, Estevez Cid F, Sanchez Dominguez E, Rios Barrera R, Sbraga F et al. Perceval Less Invasive Aortic Replacement Register: multicentric Spanish experience with the Perceval S bioprosthesis in moderate–high-risk aortic surgery. Interact CardioVasc Thorac Surg 2018;26:596–601.

22) Concistré G, Baghai M, Santarpino G, Royse A, Scherner M, Troise G, Glauber M, Solinas M.
Clinical and hemodynamic outcomes of the Perceval sutureless aortic valve from a real-world registry. Interdiscip Cardiovasc Thorac Surg. 2023;36:ivad103.

23) Vilalta V, Alperi A, Cediel G, Mohammadi S, Fernández-Nofrerias E, Kalvrouziotis D, Delarochellière R, Paradis JM, González-Lopera M, Fadeuilhe E, Carrillo X, Abdul-Jawad Altisent O, Rodríguez-Leor O, Voisine P, Bayés-Genís A, Rodés-Cabau J. Midterm Outcomes Following Sutureless and Transcatheter Aortic Valve Replacement in Low-Risk Patients With Aortic Stenosis.
Circ Cardiovasc Interv. 2021;14:e011120.

24) Suri RM, Javadikasgari H, Heimansohn DA, Weissman NJ, Ailawadi G, Ad N, Aldea GS, Thourani VH, Szeto WY, Michler RE, Michelena HI, Dabir R, Fontana GP, Kessler WF, Moront MG, Brunsting LA 3rd, Griffith BP, Montoya A, Subramanian S, Mostovych MA, Roselli EE. Prospective US investigational device exemption trial of a sutureless aortic bioprosthesis: One-year outcomes. J Thorac Cardiovasc Surg. 2019;157:1773-1782.

25) Concistrè G, Bianchi G, Margaryan R, Zancanaro E, Chiaramonti F, Kallushi E, Gasbarri T, Murzi M, Varone E, Simeoni S, Leone A, Santarelli F, Farneti P, Solinas M. Ten-year experience with sutureless Perceval bioprosthesis: single-centre analysis in 1157 implants. J Cardiovasc Med (Hagerstown). 2023;24:506-513.

26) Fischlein T, Caporali E, Asch FM, Vogt F, Pollari F, Folliguet T, Kappert U, Meuris B, Shrestha ML, Roselli EE, Bonaros N, Fabre O, Corbi P, Troise G, Andreas M, Pinaud F, Pfeiffer S, Kueri S, Tan E, Voisine P, Girdauskas E, Rega F, García-Puente J, De Kerchove L, Lorusso R. Hemodynamic Performance of Sutureless vs. Conventional Bioprostheses for Aortic Valve Replacement: The 1-Year Core-Lab Results of the Randomized PERSIST-AVR Trial. Front Cardiovasc Med. 2022;9:844876.

27) Lamberigts M, Szecel D, Rega F, Verbrugghe P, Dubois C, Meuris B. Sutureless aortic valves in isolated and combined procedures: Thirteen years of experience in 784 patients. J Thorac Cardiovasc Surg. 2024;167:1724-1732.

28) Lin CY, Lee HA, Lin PJ, Wang CL, Hung KC, Tsai FC. Sutureless aortic bioprosthesis replacement in elderly Asian patients with aortic stenosis: Experience in a single institution. Biomed J. 2018;41:265-272.

29) D'Onofrio A, Salizzoni S, Filippini C, Tessari C, Bagozzi L, Messina A, Troise G, Tomba MD, Rambaldini M, Dalén M, Alamanni F, Massetti M, Mignosa C, Russo C, Salvador L, Di Bartolomeo R, Maselli D, De Paulis R, Alfieri O, De Filippo CM, Portoghese M, Bortolotti U, Rinaldi M, Gerosa G. Surgical aortic valve replacement with new-generation bioprostheses: Sutureless versus rapid-deployment. J Thorac Cardiovasc Surg. 2020;159:432-442.

30) Muneretto C, Solinas M, Folliguet T, Di Bartolomeo R, Repossini A, Laborde F, Rambaldini M, Santarpino G, Di Bacco L, Fischlein T. Sutureless versus transcatheter aortic valves in

elderly patients with aortic stenosis at intermediate risk: A multi-institutional study. J Thorac Cardiovasc Surg. 2022;163:925-935.

31) Kim DJ, Lee S, Joo HC, Youn YN, Yoo KJ, Lee SH. Clinical and Hemodynamic Outcomes in 121 Patients Who Underwent Perceval Sutureless Aortic Valve Implantation - Early Results From a Single Korean Institution. Circ J. 2021;85:1011-1017.

32) Schizas N, Samiotis I, Nazou G, Iliopoulos DC, Anagnostopoulos I, Kousta M, Papaioannou N, Argiriou M, Dedeilias P. Perceval-S over time. Clinical outcomes after ten years of usage. J Cardiothorac Surg. 2024;19:192.

33) Berretta P, Andreas M, Meuris B, Langenaeken T, Solinas M, Concistrè G, Kappert U, Arzt S, Santarpino G, Nicoletti A, Misfeld M, Borger MA, Savini C, Gliozzi G, Albertini A, Mikus E, Fischlein T, Kalisnik J, Martinelli GL, Cotroneo A, Mignosa C, Ricasoli A, Yan T, Laufer G, Di Eusanio M. Sutureless and rapid deployment versus sutured aortic valve replacement: a propensity-matched comparison from the Sutureless and Rapid Deployment International Registry. Eur J Cardiothorac Surg. 2022;62:ezac378.

34) Kaitovic M, Micovic S, Nesic I, Raickovic T, Dotlic J, Stojanovic I, Gazibara T. An Analysis of Early Results after Valve Replacement in Isolated Aortic Valve Stenosis by Using Sutureless vs. Stented Bioprostheses: A Single-Center Middle-Income Country Experience. Medicina (Kaunas). 2023;59:1032.

35) Mujtaba SS, Ledingham S, Shah AR, Clark S, Pillay T, Schueler S. Early Clinical Results of Perceval Sutureless Aortic Valve in 139 Patients: Freeman Experience. Braz J Cardiovasc Surg. 2018;33:8-14.

36) Guner Y, Çiçek A, Karacalilar M, Ersoy B, Kyaruzi M, Onan B. Comparison of Postoperative Outcomes of Sutureless versus Stented Bioprosthetic Aortic Valve Replacement. Braz J Cardiovasc Surg. 2022;37:328-334.

37) Pollari F, Berretta P, Albertini A, Carrel T, Teoh K, Meuris B, Villa E, Kappert U, Andreas M, Solinas M, Misfeld M, Savini C, Fiore A, Shrestha M, Santarpino G, Martinelli GL, Mignosa C, Glauber M, Yan T, Fischlein T, Di Eusanio M. Pacemaker after Sutureless and Rapid-Deployment Prostheses: A Progress Report from the SURD-IR. Thorac Cardiovasc Surg. 2023;71:557-565.

38) Santarpino G, Lorusso R, Peivandi AD, Atzeni F, Avolio M, Dell'Aquila AM, Speziale G. In-Hospital Mortality and Risk Prediction in Minimally Invasive Sutureless versus Conventional Aortic Valve Replacement. J Clin Med. 2022;11:7273.

39) Raickovic T, Zivkovic I, Ragus T, Tomic S, Vukovic P, Nezic D, Peric M, Micovic S. Initial experience with the Perceval S sutureless aortic valve. Kardiochir Torakochirurgia Pol. 2020;17:20-23.

40) Paparella D, Santarpino G, Moscarelli M, Guida P, De Santis A, Fattouch K, Martinelli L, Coppola R, Mikus E, Albertini A, Del Giglio M, Gregorini R, Speziale G. Minimally invasive

aortic valve replacement: short-term efficacy of sutureless compared with stented bioprostheses. Interact Cardiovasc Thorac Surg. 2021;33:188-194.

41) Ensminger S, Fujita B, Bauer T, Möllmann H, Beckmann A, Bekeredjian R, Bleiziffer S, Landwehr S, Hamm CW, Mohr FW, Katus HA, Harringer W, Walther T, Frerker C; GARY Executive Board. Rapid Deployment Versus Conventional Bioprosthetic Valve Replacement for Aortic Stenosis. J Am Coll Cardiol. 2018;71:1417-1428.

42) Santarpino G, Lorusso R, Moscarelli M, Mikus E, Wisniewski K, Dell'Aquila AM, Margari V, Carrozzo A, Barbato L, Fiorani V, Lamarra M, Fattouch K, Squeri A, Giannini F, Marchese A, Farahani K, Gregorini R, Comoglio C, Martinelli L, Calvi S, Avolio M, Paparella D, Albertini A, Speziale G. Sutureless versus transcatheter aortic valve replacement: A multicenter analysis of "real-world" data. J Cardiol. 2022;79:121-126.

43) Jayet A, Lu H, Monney P, Verdugo-Marchese M, Gunga Z, Rancati V, Ltaief Z, Kirsch M. Thrombocytopenia among Patients Undergoing Aortic Valve Replacement Using the Sutureless Perceval S Bioprosthesis: A Retrospective Study. J Clin Med. 2024;13:1083.

44) Kim HJ, Kang DY, Park H, Ahn JM, Kim JB, Kim SO, Ok YJ, Lee SH, Pyo WK, Ko E, Lee SA, Kim DH, Park SJ, Park DW, Choo SJ. Comparison of Sutureless Bioprosthetic Valve With Surgical or TAVR for Severe Aortic Stenosis. JACC Asia. 2021;1:317-329.

45) Nakamura Y, Narita T, Kuroda M, Nakayama T, Tsuruta R, Yoshiyama D, Yasumoto Y, Sawa S, Furutachi A, Ito Y. Sutureless Aortic Valve Replacement Through Lateral Mini-Thoracotomy − Feasibility and Effectiveness. Circ J. 2022 Oct 25;86(11):1733-1739.

46) Villa E, Dalla Tomba M, Messina A, Trenta A, Brunelli F, Cirillo M, Mhagna Z, Chiariello GA, Troise G. Sutureless aortic valve replacement in high risk patients neutralizes expected worse hospital outcome: A clinical and economic analysis. Cardiol J. 2019;26:56-65.

47) Bacco LD, D'Alonzo M, Eusanio MD, Rosati F, Solinas M, Baudo M, Folliguet T, Benussi S, Fischlein T, Muneretto C. Sutureless Aortic Valve Replacement vs. Transcatheter Aortic Valve Implantation in Patients with Small Aortic Annulus: Clinical and Hemodynamic Outcomes from a Multi-Institutional Study. Braz J Cardiovasc Surg. 2024;39:e20230155.

48) Berretta P, Meuris B, Kappert U, Andreas M, Fiore A, Solinas M, Misfeld M, Carrel TP, Villa E, Savini C, Santarpino G, Teoh K, Albertini A, Fischlein T, Martinelli G, Mignosa C, Glauber M, Shrestha M, Laufer G, Phan K, Yan T, Di Eusanio M. Sutureless Versus Rapid Deployment Aortic Valve Replacement: Results From a Multicenter Registry. Ann Thorac Surg. 2022;114:758-765.

Appendix 3. Studies in the meta-regression

NUMBER	AUTHOR	YEAR	NATION	N. pts	MALE	FEMALE	Study Design	hospital mortality	F. up mortality	Lenght of Follow up	Survival	Months Survival
1	Zubarevich et al	2023	Germany	200	127	73	Retrospective single center study	9	16	12		
2	Martinez-Comedador et al.	2021	Spain	214	85	129	Retrospective single center study	1			85.7	39
3	Lam et al.	2022	US	205	122	83	Retrospective observational analysis	1	7	12		
4	Glauber M et al.	2021	Multinational	69	39	30	Multicenter registry	2	3	35		
5	Kim et al.	2018	Korea	12	5	7	Retrospective single center study	0		12	83.3	60
6	Fischlein T et al.	2021	Multinational	407	200	207	Prospective randomized sutureless vs stented	4	15	12		
7	Niinami H et al.	2023	Japan	204	76	128	Prospective observactional study	1	9	12		
8	Chung et al.	2021	Korea	66	27	39	Retrospective single center study TAVI vs Sutureless	1	2	12		
9	Ferreira et al.	2022	Portugal	196	107	89	Retrospective single center study	4			71	60
10	Lam et al.	2019	Netherlands	132	72	60	Retrospective comparing sutureless e stented	7				
11	Hong et al.	2024	Korea	113	48	65	Retrospective single center study	3			95.9	60
12	Kapadia et al.	2024	UK	132	77	55	Retrospective comparing sutureless e stented	2			95.1	48
13	Muneretto et al.	2023	Multinational	636	272	364	Retrospective multicenter study TAVI vs Sutureless	13			78.5	60
14	Ramsaransing et al.	2020	Netherlands	110	52	58	Retrospective single center study	1			97.3	12
15	Micovic et al.	2024	Multinational	328	162	166	Multicenter registry	6				
16	Bocianski et al.	2023	Poland	40	23	17	Retrospective comparing sutureless Vs mechanical	2	5	24		

17	Pollari et al.	2023	Germany	547	268	279	Retrospective single center study	18			77.5	96
18	Solinas et al	2020	Italy	503	186	317	Retrospective single center study	4			96	55
19	Muller et al.	2024	Austria	100	59	41	Retrospective single center study	5			26	36
20	Szecel et al.	2020	Belgium	468	206	262	Retrospective single center study	15	97	37		
21	Garcia et al.	2018	Spain	448	174	274	Prospective multicenter registry	20			97.9	12
22	Concistre et al.	2023	Multinational	1652	761	891	Multicenter registry	13			88.9	12
23	Vilalta et al.	2021	Canada	325	114	211	Multicenter registry TAVI vs Sutureless	7	31	24		
24	Suri et al.	2018	US	300	163	137	Prospective multicenter registry	5	15	12		
25	Concistre et al.	2023	Italy	1157	440	717	Monocenter registry	16			96.5	53
26	Fischlein T et al.	2022	Multinational	285	121	164	Randomized trial Sutureless vs Stented	2	10	12		
27	Lamberigts et al.	2024	Belgium	784	279	505	Retrospective single center study	26	68	12		
28	Lin et al.	2018	Taiwan	15	6	9	Retrospective single center study	1	1	12		
29	D'Onofrio et al.	2020	Italy	349	115	234	Retrospective comparing sutureless e rapid deployment	2				
30	Muneretto et al.	2022	Multinational	481	174	307	Register sutureless vs TAVI	10			98.9	60
31	Kim et al.	2021	Korea	107	57	50	Comparison sutureless vs TAVI	1	4	12		
32	Schizas et al.	2024	Greece	205	70	135	Single center retrospective		63	80		
33	Berretta et al.	2021	Multinational	1290	597	693	Multicenter registry	34				
34	Kaitovic et al.	2023	Serbia	101	33	68	Single center registry sutureless vs stented	2	11	24		
35	Mujtaba et al.	2017	UK	139	65	74	Single center retrospective	3				

36	Guner et al.	2022	Turkey	28	14	14	Comparison sutureless vs stented	1				
37	Pollari et al.	2022	Multinational	2604	1000	1604	Multicenter registry	57				
38	Santarpino et al.	2021	Italy	443	146	297	Multicenter registry	10				
39	Raickovic et al.	2020	Serbia	24	9	15	Retrospective monocenter	0				
40	Paparella et al.	2021	Italy	475	180	295	Multicenter registry	4				
41	Ensminger et al.	2018	Germany	900	376	524	Sutureless VS rapid deployment	25				
42	Santarpino et al.	2022	Italy	684	250	434	Multicenter registry	6				
43	Jayet et al.	2024	Switzerland	103	63	36	Retrospective monocenter	2				
44	Kim et al.	2021	Korea	121	57	65	Multicenter registry	2				
45	Nakamura et al.	2022	Japan	38	14	24	Retrospective single center study	0				
46	Villa et al.	2019	Italy	113	39	74	Monocenter registry	0				
47	Di Bacco et al.	2024	Multinational	320	29	291	Multicenter registry sutureless vs TAVI	4			88.5	36
48	Berretta et al.	2022	Multinational	2643	1225	1418	Multicenter registry	31				

Appendix 4. Quality assessment based on standardized scales for the studies included in the meta-regression

Section A. Observational studies (assessed with the Newcastle-Ottawa Scale)

NUMBER	AUTHOR	SCORE	SELECTION	COMPARABILITY	OUTCOME/EXPOSURE
1	Zubarevich et al	7	***	*	***
2	Martinez-Comedador et al.	7	***	*	***
3	Lam et al.	8	****	**	**
4	Glauber M et al.	7	***	*	***
5	Kim et al.	5	***	/	**
7	Niinami H et al.	6	***	/	***
8	Chung et al.	7	****	*	**
9	Ferreira et al.	5	***	/	**
10	Lam et al.	8	****	**	**
11	Hong et al.	6	***	/	***
12	Kapadia et al.	8	****	**	**
13	Muneretto et al.	8	****	**	**
14	Ramsaransing et al.	6	***	/	***
15	Micovic et al.	5	***	/	**
16	Bocianski et al.	8	****	*	***
17	Pollari et al.	7	***	*	***
18	Solinas et al	6	***	/	***
19	Muller et al.	6	***	/	***
20	Szecel et al.	7	***	*	***
21	Garcia et al.	6	***	/	***
22	Concistre et al.	7	***	*	***
23	Vilalta et al.	9	****	**	***

24	Suri et al.	6	***	/	***
25	Concistre et al.	5	***	/	**
27	Lamberigts et al.	6	***	/	***
28	Lin et al.	5	***	/	**
29	D'Onofrio et al.	8	****	**	**
30	Muneretto et al.	9	****	**	***
31	Kim et al.	5	***	/	**
32	Schizas et al.	7	***	*	***
33	Berretta et al.	8	****	**	**
34	Kaitovic et al.	9	****	**	***
35	Mujtaba et al.	5	***	/	**
36	Guner et al.	9	****	**	***
37	Pollari et al.	6	***	/	***
38	Santarpino et al.	8	****	*	***
39	Raickovic et al.	5	**	/	***
40	Paparella et al.	9	****	**	***
41	Ensminger et al.	9	****	**	***
42	Santarpino et al.	9	****	**	***
43	Jayet et al.	6	***	*	**
44	Kim et al.	6	***	*	**
45	Nakamura et al.	6	***	*	**
46	Villa et al.	6	***	*	**
47	Di Bacco et al.	6	***	*	**
48	Berretta et al.	7	***	**	**

Section B. Randomized Controlled Trial (assessed with the Cochrane Risk of Bias Scale)

NUMBER	AUTHOR	OVERALL RISK	RANDOMIZATIO N	INTERVENTION	OUTCOME DATA	MEASUREMENT	REPORTING
6	Fischlein T et al.	Low-moderate	Low risk	Moderate risk	Low risk	Low risk	Low risk
26	Fischlein T et al.	Low	Low risk	Low risk	Low risk	Low risk	Low risk

Appendix 5. PRISMA flowchart of study selection

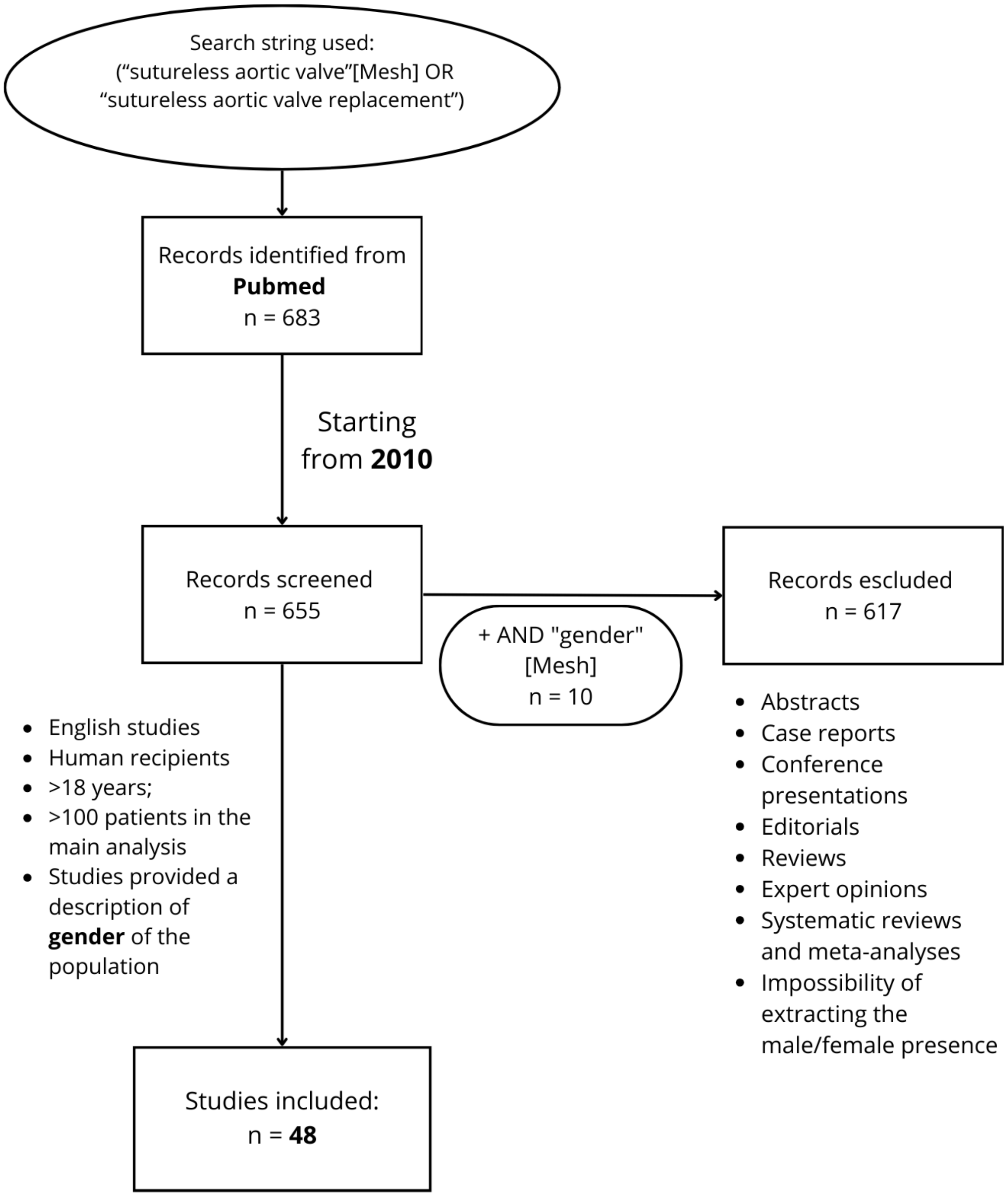

Database last accessed on July 1st, 2025
