# Supplementary material for: Sex-Related Outcome After Sutureless Aortic Valve Replacement With Perceval Plus: Results From a Global Registry and Meta-Regression
Source: Interdiscip Cardiovasc Thorac Surg. 2026 Jun 3;41(6):ivag170. doi: 10.1093/icvts/ivag170 (PMC13278764; doi:10.1093/icvts/ivag170)
Supplement: ivag170_Supplementary_Data [file ivag170_supplementary_data.zip › Supplementary file - Appendix 1.pdf]

Appendix 1. Subject disposition by site

Site ID	Site Name	City	Country	Principal Investigator	Total N=495	Pts from SURE-AVR N= 45
1124002	Université Laval	Quebec	Canada	Pierre Voisine	14	-
1124001	St. Michael's Hospital	Toronto	Canada	Gianluigi Bisleri	1	-
1124003	Foothills Medical Center	Calgari	Canada		8	-
1840004	Ochsner Clinic Foundation	New Orleans	USA	Eugene Parrino	20	
1840005	East Carolina University	Greenville	USA	Micheal Bates	3	-
1840008	Duke University	Durham	USA	G. Chad Hughes	11	-
1840009	Cleveland Clinic	Cleveland	USA	Eric Roselli	15	-
1840010	University Hospitals Cleveland Medical Center	Cleveland	USA	Cristian Baeza	27	-
1840013	St. Vincent Heart Center of Indiana	Indianapolis	USA	David Heimansohn	12	-
2056001	UZ Gent	Gent	Belgium	Thierry Bové	12	-
2056003	UZ Leuven	Leuven	Belgium	Bart Meuris	10	-
2056004	Ziekenhuis Oost Limburg	Genk	Belgium	Herbert Gutermann	12	-
2250001	CHU Poitiers	Poitiers	France	Pierre Corci	15	13
2276002	Klinikum Nürnberg	Nürnberg	Germany	Francesco Pollari	15	6
2276003	Universitätsklinikum Magdeburg	Magdeburg	Germany	George Awad	7	-
2276005	Herzzentrum Dresden Universitatsklinik	Dresden	Germany	Utz Kappert	21	-
2276006	Klinikum Oldenburg GGMBH AoR	Oldenburg	Germany	Friedrich Mellert	16	1
2276008	Universitätsklinikum Essen	Essen	Germany	Sharaf-Eldin Shehada	52	-
2276010	Universitätsklinikum Augsburg	Ausburg	Germany	Evaldas Girdauskas	6	-
2380002	Fondazione Poliambulanza Istituto Ospedaliero	Brescia	Italy	Giovanni Troise	12	-
2380003	Ospedale del Cuore di Massa	Massa	Italy	Marco Solinas	21	-
2380008	Ospedale Ca' Foncello di Treviso	Treviso	Italy	Giuseppe Minniti	10	-
2380009	Az. Ospedaliero-Universitaria "Ospedali Riuniti" di Trieste	Trieste	Italy	Enzo Mazzaro	5	1
2380010	A.O.U. Città della Salute e della Scienza di Torino - Ospedale Molinette	Torino	Italy	Mauro Rinaldi	6	-
2380011	Policlinico S.Orsola-Malpighi	Bologna	Italy	Davide Pacini	5	-
2380013	Policlinico Paolo Giaccone	Palermo	Italy	Vincenzo Argano	7	-
2380014	Azienda ospedaliera dei Colli - Ospedale Monaldi	Napoli	Italy	Michele Torella	15	-
2528003	Catharina Ziekenhuis	Eindhoven	The Netherlands	Kayan Lam	7	-
2620001	Hospital de Santa Maria Lisbon	Lisbon	Portugal	Angelo Nobre	49	24
2688001	Dedinje Cardiovascular Institute	Belgrade	Serbia	Slobodan Micovic	37	-
2756002	Inselspital, Universitätsspital Bern	Bern	Switzerland	Matthias Siepe	3	-
2826005	King's College Hospital	London	UK	Max Baghai	12	-
3410001	Yonsei University Severance Cardiovascular Hospital	Seoul	South Korea	Seung Hyun Lee	9	-
3410002	Seoul National University Hospital	Seoul	South Korea	Jae Woong Choi	30	-
3410004	Pusan National University Yangsan Hospital	Pusan	South Korea	Hyung Gon Je	30	
	**TOTAL**				**535**	**45**
