# Supplementary material for: Sex-Related Outcome After Sutureless Aortic Valve Replacement With Perceval Plus: Results From a Global Registry and Meta-Regression
Source: Interdiscip Cardiovasc Thorac Surg. 2026 Jun 3;41(6):ivag170. doi: 10.1093/icvts/ivag170 (PMC13278764; doi:10.1093/icvts/ivag170)
Supplement: ivag170_Supplementary_Data [file ivag170_supplementary_data.zip › Supplementary file - Appendix 6, 7, 8 (Statistics).docx]

# **Appendix 6.** LS-Means analysis of LVEF changes over time in males and females.

In the later stages of follow-up, statistically significant improvements in LVEF were observed from baseline in both male and female participants

At 3 years, both sexes showed an increase compared to baseline, but only males reached statistical significance (males: LS Means estimate = 6.0943, p = 0.0015; females: LS Means estimate = 3.2750, p

= 0.0671).

At 4 years, both groups demonstrated a statistically significant increase compared to the baseline (males: LS Means estimate = 10.9044, p < 0.0001; females: LS Means estimate = 8.1954, p = 0.0012).

These findings suggest a sustained trajectory of LVEF improvement over time, particularly becoming more pronounced after 3 years.


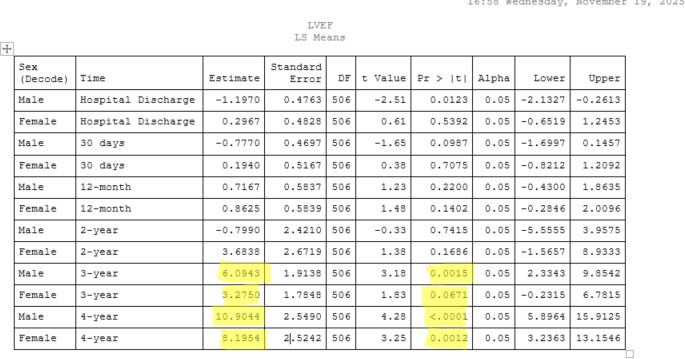


There were no statistically significant differences in LS-Means changes between males and females at any time point, except at hospital discharge (LS Means difference estimate = -1.4937, p = 0.0285).


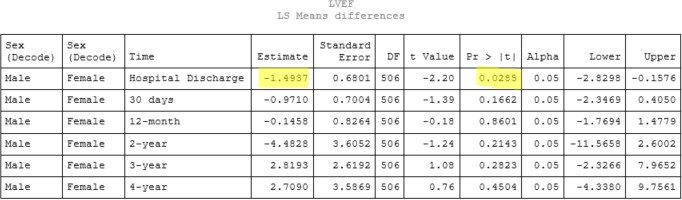


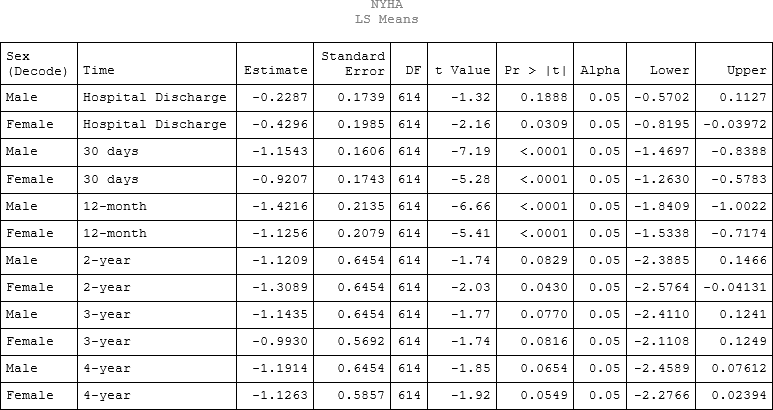


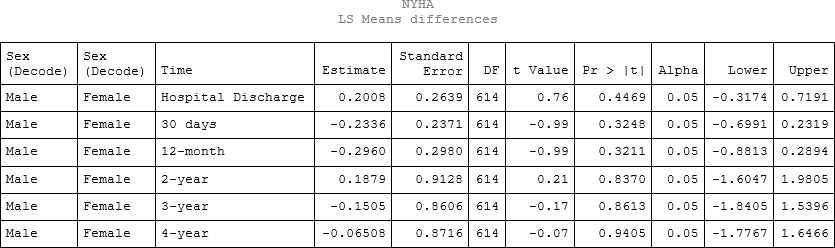


**Appendix 7**. Primary unadjusted mixed-effects models for longitudinal outcomes.

| Outcome | N included | Sex p-value | Visit p-value | Sex × Visit p-value |
| --- | --- | --- | --- | --- |
| NYHA | 489 | 0.8234 | <0.0001 | 0.8271 |
| EQ-5D (VAS) | 425 | 0.2461 | <0.0001 | 0.7850 |
| KCCQ | 430 | 0.0110 | <0.0001 | 0.6466 |
| MPG | 442 | 0.3865 | 0.0049 | 0.2261 |
| PPG | 407 | 0.5152 | 0.0021 | 0.1198 |
| EOA | 132 | 0.1116 | 0.5600 | 0.2496 |
| LV Mass | 124 | 0.6727 | 0.6316 | 0.6428 |

**Appendix 8.** Effect of CABG in adjusted mixed-effects models.

Estimates represent the effect of CABG derived from mixed-effects models adjusted for age, coronary artery disease, pulmonary hypertension, smoking status, surgical approach and concomitant procedures.

| **Outcome** | **Covariate** | **Estimate** | **SE** | **p-value** |
| --- | --- | --- | --- | --- |
| NYHA | CABG | -0.07296 | 0.3440 | 0.83 |
| EQ-5D (VAS) | CABG | 2.6818 | 2.7046 | 0.32 |
| KCCQ | CABG | 3.2083 | 3.4629 | 0.36 |
| MPG | CABG | 0.04355 | 0.7706 | 0.96 |
| PPG | CABG | 0.4134 | 1.3946 | 0.77 |
| EOA | CABG | 0.06300 | 0.1524 | 0.68 |
| LV Mass | CABG | -11.6961 | 16.2982 | 0.47 |

Abbreviations: **NYHA** - New York Heart Association; **EQ-5D (VAS)** - EuroQol 5-Dimensions questionnaire (Visual Analogue Scale); **KCCQ** - Kansas City Cardiomyopathy Questionnaire; **MPG** - Mean Pressure Gradient; **PPG** - Peak Pressure Gradient; **EOA** - Effective Orifice Area; **LV Mass** - Left Ventricular Mass.
